# Biomechanical Stress Analysis of Platform Switch Implants of Varying Diameters on Different Densities of Bone

**DOI:** 10.1155/2022/5972259

**Published:** 2022-02-24

**Authors:** Sandipan Mukherjee, Shobha Rodrigues, Mahesh M, Thilak Shetty, Umesh Pai, Sharon Saldanha, Puneeth Hedge, Vignesh Kamath, Ann Sales, Prashanth Bajantri

**Affiliations:** Department of Prosthodontics, Manipal College of Dental Sciences, Mangalore, Manipal Academy of Higher Education, Manipal, Karnataka, India

## Abstract

**Purpose:**

The purpose of this study was to evaluate and compare the strain developed in D2 and D3 types of bones on vertical loading by platform switch implants of different diameters.

**Materials and Methods:**

Implants of diameters 3.25 mm, 4.2 mm, and 5.0 mm and of length 11.5 mm were taken and placed each on D2 and D3 bone models. Strain gauges were attached on the buccal and the lingual sides on each of these samples, and a vertical load of 190N was placed on the samples. The strain was recorded using a data logger. The data obtained was analysed using one-way ANOVA and post hoc Tukey test.

**Results:**

In D2 and D3 bone models, 3.25 mm significantly showed greater bone strain values. The buccal side strain was higher irrespective of the implant diameter and density of bone.

**Conclusion:**

Within the limitations of the study, it may be concluded that the narrow diameter implant produces greater strain than 4.2 and 5.0 mm diameter implants, respectively. The buccal side consistently produced higher bone strain values.

## 1. Introduction

The success of prosthesis is assessed by the marginal bone level around the implant. Marginal bone loss is a complicated, multifactorial effect of mechanical and biological factors.

Biomechanical factors that influence stress in the bone around an implant include the quality and quantity of bone, implant design, diameter, and platform switching among other factors [1]. Misch classified bone quality into 4 types, based on their densities: D1, D2, D3, and D4.

However, D2 and D3 are the most commonly found types of bones and are the most suited for osseointegration [2]. The bone quality in the planned area dictates outcome of the treatment, implant design, surgical approach, time of healing, and initial progressive bone loading during prosthetic reconstruction [3, 4].

Platform switching has been found to decrease or remove any predictable postrestoration remodeling of bone at the crest. The concept was introduced by Lazzara and Porter, who showed minimal vertical bone loss radiographically around implants with mismatched abutment [5]. According to biomechanical theory, connection of the implant with an abutment which is smaller in diameter restricts resorption of bone by guiding occlusal forces in the same direction as the axis of implant and by transferring away stress concentration zone from crestal bone implant surface [6].

Two types of loads, namely, vertical and transverse, from mastication, act on implant-supported prosthesis leading to axial forces and bending moments leading to concentration of stress on both implant and the bone [7, 8]. Hence, qualification and quantification of these forces can help to determine the clinical behavior of these devices [7]. Photo elasticity, finite element analysis (FEA), and strain measurement techniques have all been used to determine the developed stress around the implants. The FEA results concluded with the decrease in implant diameter, and there was an increase in the stress concentration. Abutment connection design was also found to be responsible for the concentration of strain in bone around the implant. However, little is known about the stress variation with varying bone densities in this regard [9].

Wagenberg and Froum conducted a 11-year prospective study to evaluate implant survival and crestal bone levels around implants that used platform switching concept. This is the longest follow-up prospective investigation and confirms the concept of preservation of crestal bone [10]. The configuration of platform switched abutments helped produce significantly less strain concentration in the peri-implant bone and provides a damping effect. Rodriguez et al. by finite element analysis compared the biomechanical response of three types of implant-abutment configurations both before and after establishment of new biological width. The two implant-abutment designs featuring a smaller diameter abutment on a larger diameter implant platform achieved better results than the implant featuring implant platform and abutment of the same-diameter, even though their initial biomechanical load potential was lower [11].

Chun et al. investigated the behaviors of reduced-platform restorations using 3-dimensional FEA and concluded that there was a significant reduction in the stress at the implant bone interface by 10% [12].

Hence, in this study, the authors aimed at analyzing and comparing the stress distribution in comparable models of different densities of bone with platform switched abutments with varying diameters implants keeping the length constant. The null hypothesis was that the stress distribution was not affected by different densities of bone and diameter of implants when platform switched abutments are used.

## 2. Materials and Methods

Following Institutional Ethical Committee clearance and obtaining appropriate consent, this study was carried out in the Department of Prosthodontics of the Institution, Mangalore, India, in collaboration with Konkan Speciality Polyproducts Pvt. Ltd. (Baikampady Industrial Area, Mangalore, India).

The study was designed to be conducted on two different types of bone: D2 and D3 simulators. The American Society for Testing Materials (ASTM) has standardized polyurethane blocks for testing orthopedic implants as they have same mechanical properties as cancellous bone [13]. For this reason, the polyurethane blocks of different densities, simulating the D2 and D3 types of bones, were selected to perform this study. Density of these bones used in this study is in accordance with Misch's classification.

### 2.1. Polyurethane Blocks

Two trabecular bone models of sawbones were prepared, one corresponding to D2 and the other D3 types of bones in accordance with bone density classification of Misch. For D2, a solid polyurethane block of 40pcf (pound force per cubic foot) of dimensions 2 × 2 × 5 cm was prepared (model 1522-05; Pacific Research Laboratories) and a 3-mm-thick commercially available synthetic cortical shell (model 3401-02; Pacific Research Laboratories) was attached over it [14]. The D3 bone was simulated using a polyurethane block of 10 pcf of the same dimensions (model 1522-01; Pacific Research Laboratories) according to the manufacturer's instructions. Three specimens of artificial bones were prepared for each diameter of implants.

### 2.2. Preparation of Sample

**Figure 1:**
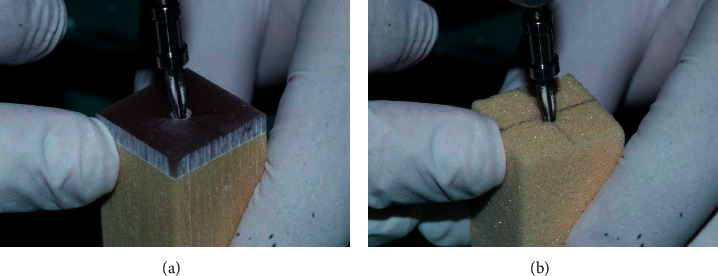
Sequential osteotomy preparation for the placement of implants representative polyurethane blocks of 40 pcf of dimension 2 × 2 × 5 cm with 3 mm cortical shell simulating d2 bone (a) and representative polyurethane blocks of 10 pcf of dimension 2 × 2 × 5 cm d3 bone (b).

Sequential osteotomy was done on D2 and D3 bone, and implants of diameters each 3.25 mm, 4.2 mm and 5.0 mm were placed, respectively, using Paltop surgical kit (Paltop Master with drill stops kit) (Figures 1(a) and 1(b)).

Then, the respective platform switched abutments were placed onto the implants.

Strain gauges (Rosette strain gauges, National instrument engineering, Jaipur, India) were attached on the buccal and on the lingual sides at the implant abutment junction (IAJ) to measure the strain, which was connected to a display unit attached with a computer.

### 2.3. Study Protocol

The samples were placed on a universal testing machine (Z020; Zwick Roell), Konkan Speciality Polyproducts Pvt. Ltd. (Baikampady Industrial Area, Mangalore).

The testing machine was connected to a computer, while the strain gauges were connected to custom made data logger (National Instrument Engineering, Jaipur, India) which recorded the data separately on buccal and lingual when subjected to load. The samples were subjected to a vertical load of 190N on the abutment at a head speed of 1 mm/min.

Three recordings were taken for each specimen, and the average was obtained for each.

## 3. Results

The strains were measured at buccal and lingual sides under vertical loading.

Strain generated at implant abutment junction in 3 different diameters of platform switched implants on D2 and D3 types of bones was compared using one-way ANOVA and post hoc Tukey test (Tables 1 and 2).

**Figure 2:**
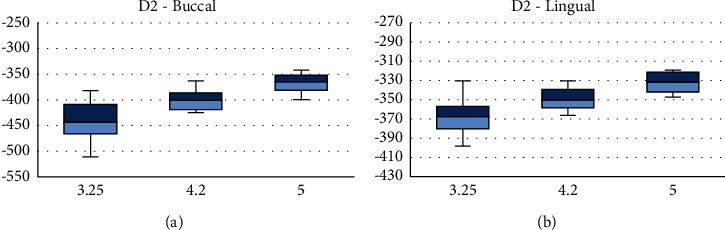
Medians and interquartile ranges of peak values of principal microstrains on buccal (a) and lingual (b) sides of D2 bone under vertical loading.

Under vertical loading, on D2, the highest strain was observed with 3.25 mm, on the buccal, while 4.2 mm produced the highest strain on the lingual side (Figures 2(a) and 2(b)).

**Figure 3:**
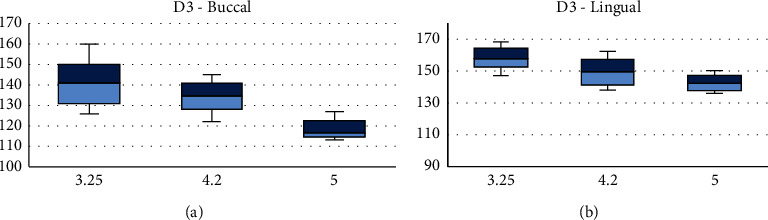
Medians and interquartile ranges of peak values of principal microstrains on buccal (a) and lingual (b) sides of d3 bone under vertical loading.

Under vertical loading on D3, the highest strain was observed with 3.25 mm on both the buccal and lingual (Figures 3(a) and 3(b)).

A comparison between the groups shows that, for each diameter, the buccal strain is significantly more (*p* < 0.001) than the lingual strain irrespective of diameter and density of bone.

## 4. Discussion

There have been many studies conducted previously which concluded that the platform switching was effective in reducing the crestal bone loss [15]. They compared mainly with the conventional and platform switching, but none, to the best of author's knowledge, compared the effect of various diameters of platform switch implants on different types of bones. Hence, it becomes imperative to analyze the distribution of stress/strain and its relation with the different implant and abutment connection and bone. The current study attempted to analyze the amount of stress distribution amongst three different diameters of platform switch implants on two most commonly found bone types: D2 and D3, as various studies previously.

The present study found that 3.25 mm diameter platform switched implants produce significantly more bone strain values, *p* < 0.01 (buccal: 444; lingual: 367.5) when compared to 4.2 mm (buccal: 402; lingual: 350) and 5.0 mm (buccal: 365; lingual: 331.5) on D2 and 3.25 mm diameter platform switched implants produces significantly more bone strain values (buccal: 141; lingual: 157) when compared to 4.2 mm (buccal: 134.5; lingual: 149.5) and 5.0 mm (buccal: 116; lingual: 142.5) on D3 bone. The results also indicate greater bone strain values on the buccal cortical plate as compared to the lingual cortical plate irrespective of the implant diameter and density of bone. This is may be due to the fact that buccal cortical plate is relatively thinner when compared to the lingual cortical plate [16]. The results also indicate greater bone strain values in the D2 type of bone as compared to the D3 type of bone. This may be explained by the quality and internal architecture of D2 and D3 types of bone. The density of D2 bone is in the range of 850 to 1250 Hounsfield units, and density of D3 is 350 to 850 Hounsfield units. The stress dissipating characteristics of D3 bone on account of its higher trabecular content is responsible for lower bone strain values. Dental implant complications develop through fracture of the porcelain, loosening of the screw, implant component fracture, and crestal bone loss [17–20]. These complications arise often due to incorrect loading of the implant prosthesis complex, which results in the undue stresses in the different components of the dental implants and bone [21].

Histological studies have been of great help in further stressing on the beneficial effect of platform switch implants [22]. Though many studies analysed the stress distribution on the bone around the implants, no studies, to the best knowledge of the author, examined the effect of various diameters of platform switched implants on the various densities of peri-implant bone at the implant abutment complex. There was a need for different method to measure the strain to relatively get the values closer to real time other than FEA. Hence, strain gauges became quite popular as a testing aid along with the universal testing machine.

One of the possible reasons is a change of design of platform switch implants that increases the space between crestal bone and the inflammatory cell infiltrate in the microgap reducing or preventing any chances of bone loss due to remodeling of the bone [8, 23]. Some researchers also suggested a shift in the stress concentration to the more cancellous bone when the implant is being loaded, thereby decreasing the chances of marginal bone loss [24–27].

Pellizzer et al. studied the influence of platform switching on stress and concluded by photoelastic analysis that wide diameter implants and platform switching implants had similar stress concentrations which were lower than the conventional regular diameter implants [28]. The current study found similar results that, with an increase in the diameter, there is a decrease in the stress concentration. Hsu et al. also concluded that stress in bone was reduced by increased implant diameter rather than by the platform-switching technique for immediately loaded implant [14]. Recent studies provide further insight into additional factors that may also influence peri-implant bone. Guarnieri et al. in their study reported replacement of different prosthetic components can lead to loss of bone [29]. In addition, peri-implant tissues represent a higher proinflammatory state which may contribute to bone loss. Also, laser microtextured implant surfaces and their distinct relation with surrounding tissues and marginal gingival interface may create environment changes that could modify the production of cytokines, thereby associated with protection against bacterial pathogens during the postsurgical healing phase [30]. Therefore, these additional factors promoting peri-implant bone loss needs to be investigated.

The null hypothesis was rejected as, with the increased diameter of the platform switching implants, there was a decrease in the stress around implant abutment junction irrespective of the bone type and architecture. However, further investigations are required to confirm the findings.

This study, though, tried every possible measure to simulate the clinical situation more closely, but there are few limitations to the present study. The bone simulation was done using artificial substitute. The substitute morphology and homogeneity are a limitation as the bone may not always present with the exact specifications, and this could in turn may affect the results. The authors could not simulate the dynamic nature of human mastication. The values were obtained for the areas only where the strain gauges were attached. The viscoelasticity and muscle attachments were not simulated, which is a matter of investigation and could influence the results.

## 5. Conclusion

Within the limitations of the study design, it can be concluded that 3.25 mm diameter implant produces higher strain values on D2 and D3 bones as compared to 4.0 mm and 5.0 mm diameters of implants, respectively. The buccal side had more bone strain values when compared with the lingual.

## Figures and Tables

**Table 1:** Microstrain values on vertical loading on D2 bone on buccal and lingual for different diameters of implants.

Location	Implants	Vertical loading microstrain
Buccal	3.25	−444
	4.2	−402
	5.0	−365

Lingual	3.25	−367.5
	4.2	−350
	5.0	−331.5

**Table 2:** Microstrain Values on Vertical Loading on D3 bone on Buccal and Lingual For Different Diameters of Implants.

Location	Implant	Vertical loading microstrain
Buccal	3.25	141
	4.2	134.5

Lingual	3.25	157.5
	4.2	149.5
	5.0	142

## Data Availability

Data are available from the corresponding author upon request.
